# Association of Multimorbidity With Frailty in Older Adults for Elective Non-Cardiac Surgery

**DOI:** 10.7759/cureus.15033

**Published:** 2021-05-14

**Authors:** Phui Sze Angie Au Yong, Eileen Yi Lin Sim, Collin Yih Xian Ho, Yingke He, Charlene Xian Wen Kwa, Li Ming Teo, Hairil Rizal Abdullah

**Affiliations:** 1 Division of Anaesthesiology and Perioperative Medicine, Singapore General Hospital, Singapore, SGP

**Keywords:** frailty, multimorbidity, geriatric, surgery, comorbidity, older adult, elective surgical procedures, frail cases

## Abstract

Introduction

Frailty is associated with adverse surgical outcomes. While existing studies describe the prevalence of multimorbidity and frailty in the community, the surgical population may have more severe disease and significant surgical stress. This study aims to describe the distribution of frailty and multimorbidity in the older surgical population and examine if specific comorbidities are more strongly associated with frailty.

Methods

This is a single-centre retrospective cohort study using an electronic database in the preoperative evaluation clinic, conducted in Singapore General Hospital, Singapore. All patients above 70 years old going for elective non-cardiac surgery were included. Demographics and comorbidities were analysed for their association with frailty according to the Edmonton Frail Scale.

Results

A total of 1396 out of 1398 patients were analyzed. The overall incidence of frailty was 27.8% and multimorbidity was 63.4%. Factors independently associated with frailty were age (adjusted Odds Ratio [aOR] = 1.07), female gender (aOR = 1.67), type 2 diabetes mellitus (aOR = 1.69), chronic kidney disease (aOR = 1.47), end-stage renal failure (aOR = 3.58), history of cerebrovascular accident or transient ischemic attack (aOR = 1.87), moderate anaemia (aOR = 2.11), dementia (aOR = 6.38), depression (aOR = 3.82), and peptic ulcer disease (aOR = 1.98). The presence of multi-morbidity was significantly associated with frailty, with overall increasing strength of association.

Conclusion

As the number of comorbidities increases, the odds of frailty increase. Only a small proportion of those with multimorbidity accumulate enough biological deficits to develop frailty, putting them at higher risk than with solely multimorbidity or frailty. Dementia and depression are comorbidities with strong associations that have yet to see coordinated interventional efforts in the preoperative setting.

## Introduction

Surgery places significant stress on the human body and induces major physiological responses. Frail individuals have a reduced ability to maintain homeostasis under physiological stress due to the composite effect of multiple organ deficiencies [1]. Frailty has been shown to predict adverse surgical outcomes, including serious complications, prolonged length of stay, and long-term mortality [2-4]. The etiology of frailty can be viewed as an accumulation of biological, social, and functional deficits over time. While these deficits generally accumulate with age, different individuals accumulate them at different trajectories and chronological age does not necessarily reflect the degree of frailty [5].

Due to the accumulation of biological deficits, chronic diseases may also occur concomitantly with frailty. The presence of two or more chronic diseases is known as multi-morbidity, which is associated with decreased quality of life, functional decline, and increased healthcare utilization [6]. A systematic review estimated the pooled prevalence of multi-morbidity at 33.1% worldwide, with the prevalence being 37.9% in high-income countries and 29.7% in low- to middle-income countries [7]. For comparison, a local study amongst community dwellers in Singapore estimates its prevalence at 26.2% and found that it was associated with increasing age, female gender, lower socioeconomic status, and an increasing number of mental disorders [8].

There is a certain overlap between frailty and multi-morbidity. Frailty may predispose individuals to develop chronic diseases, but can, in turn, stem from multi-morbidity. Another recent systematic review examined nine studies, pooling data from 14704 community-dwellers [9]. Out of 1271 (12%) frail patients, 868 (6%) had multi-morbidity. These 868 patients with both multi-morbidity and frailty comprised 42% of those who had multi-morbidity. While there are existing studies describing the prevalence of multi-morbidity and frailty in the community setting, there is a knowledge gap on the correlation between multi-morbidity and frailty in the surgical population, who may have more severe disease presentation and experience significant surgical stress [10-16]. This is relevant due to the increasing number of non-cardiac surgeries performed in an aging population. Improved understanding of the characteristics of those with frailty, multi-morbidity, or both will help to tailor efforts for perioperative care [17].

We hypothesize that amongst older patients requiring surgery, the presence of multi-morbidity is positively associated with the degree of frailty. This study aims to describe the distribution of frailty and multi-morbidity in the older surgical population and examine if specific comorbidities are more strongly associated with frailty than others. Our secondary aim is to look at the contribution of the individual frailty domains within Edmonton Frail Scale among frail patients.

## Materials and methods

Study design

This is a single-centre, retrospective cohort study conducted in Singapore General Hospital, a 1700-bedded academic hospital. Singhealth Institutional Review Board (Singhealth CIRB 2014/651/D) approved the study and waived the requirement for individual informed consent.

**Figure 1:**
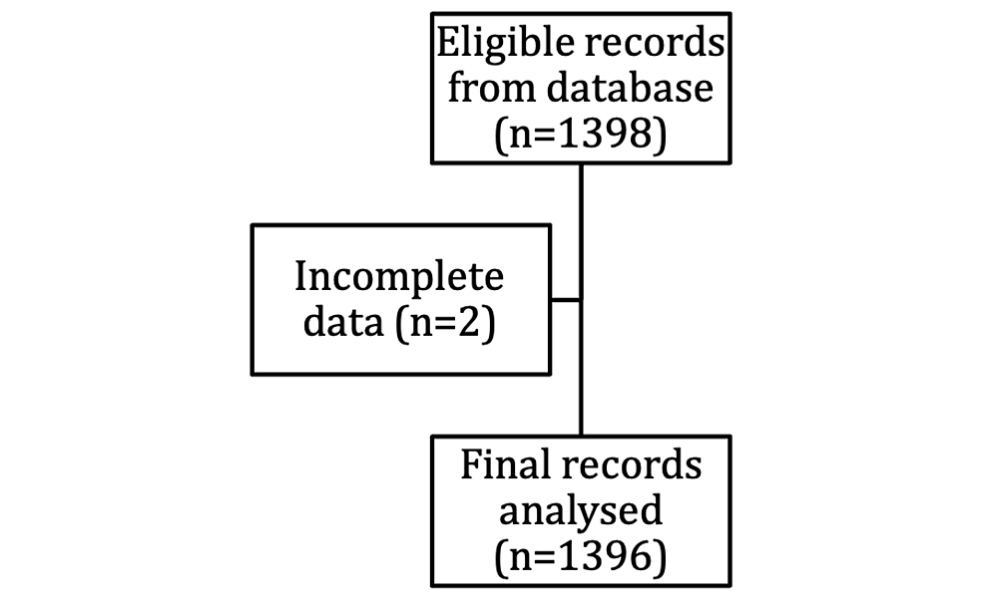
Flowchart for data collection

Our institution’s electronic clinical information system (Sunrise Clinical Manager [SCM], Allscripts, IL, USA) was accessed retrospectively for data collection (Figure 1). Data obtained include sociodemographic details, nature of the surgery, comorbidities included and as defined in Charlson’s comorbidity index, and other comorbidities not included, such as anaemia, hypertension (HTN), and chronic obstructive pulmonary disease (COPD) [18]. Multimorbidity was defined as having two or more comorbidities. Anaemia was defined according to the World Health Organisation classification: mild (haemoglobin [Hb] <12g/dL in females and <13g/dL in males), moderate (Hb <11g/dL) and severe (Hb <8g/dL). Blood investigations recorded were haemoglobin level (g/dL) and albumin (mmol/L).

Currently, there is no gold standard frailty measure and multiple classifications exist [19]. Our institution uses the Edmonton Frail Scale (EFS) as a surrogate marker of frailty for surgical risk stratification as it is a validated performance-based tool designed for rapid assessment of geriatric patients in the primary care and surgical setting by individuals with little medical training [20]. Its advantages include objective questions, easy implementation, and good correlation with clinical outcomes.

The EFS is administered as part of routine preoperative anesthesia assessment by trained nurses in the Preoperative Evaluation Clinic (PEC). The EFS consists of nine domains: two domains (cognition and functional performance) require the patient to perform activities that reflect cognition, balance, and gait. The remaining seven domains are self-reported by the patient: mood, functional independence, medication use, social support, nutrition, health attitude, continence, the burden of medical illness, and quality of life. The scoring is from 0 to 17, divided into five categories: fit (EFS 0-3), vulnerable (4-5), mildly frail (EFS 6-7), moderately frail (EFS 8-9), and severely frail (EFS >9).

The study’s inclusion criteria were all patients aged 70 and above coming for elective non-cardiac surgery seen at the PEC between January and June 2018 (n=1398). Those with incomplete data (n=2) were excluded from the final analysis. There were no duplicates. A cut-off age of 70 years old (which is higher than the World Health Organisation definition of elderly at 65 years old) was chosen to better reflect our target population. This is because Singapore has one of the highest life expectancies worldwide. According to national statistics in 2020, the average life expectancy at 65 years of age is 21.3 years. Around 14.4% of the population is above 65 years old, of whom 30% are still in the labour force, reflecting their physical fitness grossly.

Statistical analysis

Statistical analysis was performed using IBM SPSS Statistics v21.0 (IBM Corp., Armonk, NY, USA). Descriptive statistics were used to describe the baseline demographics of the whole cohort. The proportion of categorical variables was compared using the Chi-square test, while mean values of continuous variables were compared using the one-way analysis of variance (ANOVA).

Multivariable regression was performed on variables that were significant on either ANOVA or Chi-square test to assess the independent association of patient factors and comorbidities with frailty (EFS score 6 and above). A p-value of <0.05 was used to determine statistical significance.

## Results

Demographics

A total of 1396 patients were included in the analysis. Demographics by frailty classification according to EFS categories are listed in Table 1. For statistical analysis, the non-frail group consisting of fit and vulnerable patients (EFS 0-5) were analysed in comparison to the frail group consisting of mildly-frail (EFS 6-7), moderately-frail (EFS 8-9), and severely frail (EFS >9) patients. There were significant differences in baseline characteristics and comorbidities between the frail and non-frail groups. The mean age in the frail group was significantly higher at 77.3±5.2 years old (p<0.001) compared to 75.2±4.4 years old in the non-frail group. There was also a significantly higher proportion of females (31.2% vs 23%, p=0.002) in the frail group. The mean albumin level was significantly lower in the frail group at 37.5±5.5 mmol/L (p<0.001) compared to 39.2±5.6 mmol/L in the non-frail group.

**Table 1:** Demographics and baseline characteristics of the participants BMI: body mass index; DM: diabetes mellitus; CKD: chronic kidney disease; ESRF: end-stage renal failure; IHD: ischemic heart disease; OSA: obstructive sleep apnea; COPD: chronic obstructive pulmonary disease; PVD: peripheral vascular disease; CVA: cerebrovascular accident; TIA: transient ischemic attack

Variables	Total (N=1396)	Frail (N=388)	Non-frail (N=1008)	p-value
Age (year), mean (SD)	75.8 (4.7)	77.3 (5.2)	75.2 (4.4)	< 0.001
Gender, n (%) Female	737 ( 52.8 )	231 ( 59.5 )	506 ( 50.2 )	0.002
Male	659 ( 47.2 )	157 ( 40.5 )	502 ( 49.8 )	
Race, n (%) Chinese	1248 ( 89.4 )	350 ( 90.2 )	898 ( 89.1 )	0.609
Non-chinese	148 ( 10.6 )	38 ( 9.8 )	110 ( 10.9 )	-
BMI in kg/m^2^, mean (SD)	24.5 (4.2)	24.3 (4.6)	24.6 (4)	0.13
DM, n (%)	429 ( 30.7 )	166 ( 42.8 )	263 ( 26.1 )	< 0.001
DM on insulin, n (%)	49 ( 3.5 )	26 ( 6.7 )	23 ( 2.3 )	< 0.001
Liver disease, n (%)	81 ( 5.8 )	19 ( 4.9 )	62 ( 6.2 )	0.441
Albumin in mmol/L, mean (SD)	38.6 (5.6)	37.5 (5.5)	39.2 (5.6)	< 0.001
Malignancy, n (%)	474 ( 34 )	131 ( 33.7 )	343 ( 34 )	0.976
CKD, n (%)	224 ( 16 )	103 ( 26.5 )	121 ( 12 )	< 0.001
ESRF, n (%)	56 ( 4 )	41 ( 10.6 )	15 ( 1.5 )	< 0.001
IHD, n (%)	301 ( 21.6 )	111 ( 28.6 )	190 ( 18.8 )	< 0.001
Hypertension , n (%)	1064 ( 76.2 )	328 ( 84.5 )	736 ( 73.0 )	< 0.001
Congestive heart failure, n (%)	33 ( 2.4 )	17 ( 4.4 )	16 ( 1.6 )	0.004
OSA, n (%)	19 ( 1.4 )	7 ( 1.8 )	12 ( 1.2 )	0.53
COPD, n (%)	54 ( 3.9 )	21 ( 5.4 )	33 ( 3.3 )	0.155
Asthma, n (%)	40 ( 2.9 )	7 ( 1.8 )	33 ( 3.3 )	0.159
Pulmonary hypertension, n (%)	2 ( 0.1 )	0 ( 0 )	2 ( 0.2 )	-
PVD, n (%)	60 ( 4.3 )	32 ( 8.2 )	28 ( 2.8 )	< 0.001
History of CVA/TIA, n (%)	118 ( 8.5 )	52 ( 13.4 )	66 ( 6.5 )	< 0.001
Dementia, n (%)	30 ( 2.1 )	22 ( 5.7 )	8 ( 0.8 )	< 0.001
Depression, n (%)	25 ( 1.8 )	16 ( 4.1 )	9 ( 0.9 )	< 0.001
Hemiplegia, n(%)	14 ( 1 )	10 ( 2.6 )	4 ( 0.4 )	0.001
Connective tissue disease, n (%)	43 ( 3.1 )	13 ( 3.4 )	30 ( 3.0 )	0.85
Peptic ulcer disease, n (%)	58 ( 4.2 )	29 ( 50 )	29 ( 50 )	< 0.001
Hemoglobin in g/dL, mean (SD)	12.8 (1.7)	12.2 ( 7.5 )	13 ( 3.4 )	< 0.001
Mild anaemia, n (%)	317 ( 22.7 )	114 ( 29.4 )	203 ( 20.1 )	< 0.001
Moderate anaemia, n (%)	176 ( 12.6 )	80 ( 20.6 )	96 ( 9.5 )	< 0.001
Severe anaemia, n (%)	6 ( 0.4 )	4 ( 1.0 )	2 ( 0.2 )	-

**Figure 2:**
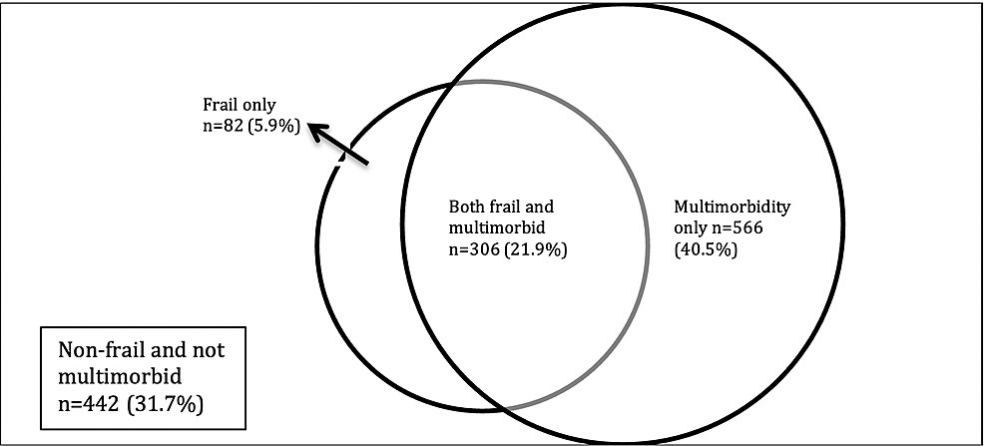
Distribution of multi-morbidity and its overlap with frailty

The overall incidence of frailty, as defined by EFS >6, was 27.8% (n=388). The median EFS score in our study population was 4 (interquartile range=2-6, range=0-13). The incidence of multi-morbidity was 63.4%. Figure 2 shows the distribution of patients with solely multi-morbidity or frailty or a combination of both. A total of 306 patients (21.9%) presented with multi-morbidity and frailty, 82 (5.9%) presented with only frailty, and 566 (40.5%) presented with only multi-morbidity. 

Analyzing by surgical discipline, the highest percentage of frail (55.4%) and multimorbid (85.1%) patients were admitted to Vascular Surgery, followed by Urology (28.3% frail, 35.7% multimorbid). The General Surgery discipline had a higher percentage of patients with multi-morbidity (73.2%) relative to Orthopaedics (45.9%). Patients admitted to both of these two disciplines had similar percentages of frailty (25.5% vs 24.8%).

Using univariate logistic regression (Table 2), there was a significant association of these comorbidities with being frail: diabetes mellitus (DM), DM on insulin treatment, chronic kidney disease (CKD), end-stage renal failure (ESRF), ischemic heart disease (IHD), hypertension (HTN), congestive heart failure (CHF), peripheral vascular disease (PVD), cerebrovascular accident/transient ischemic attack (CVA/TIA), dementia, depression, hemiplegia, peptic ulcer disease (PUD), and anaemia.

**Table 2:** Association of comorbidities with being frail

Variables	Unadjusted Odds Ratio (95% CI)	p-value	Adjusted Odds Ratio (95% CI)	p-value
Age	1.09 (1.07, 1.12)	<0.001	1.07 (1.04, 1.11)	< 0.001
Female Gender	1.45 (1.15, 1.85)	0.002	1.67 (1.25, 2.22)	< 0.001
Weight	0.98 (0.97, 0.99)	0.001	0.99 (0.98, 1.01)	0.261
Diabetes mellitus	2.12 (1.66, 2.71)	<0.001	1.69 (1.28, 2.24)	< 0.001
Chronic kidney disease	2.65 (1.97, 3.56)	<0.001	1.47 (1.03, 2.09)	0.034
End-stage renal failure	7.82 (4.37, 14.75)	<0.001	3.58 (1.79, 7.49)	< 0.001
Mild anaemia	2.09 (1.58 2.76)	<0.001	1.8 (1.32, 2.46)	< 0.001
Moderate anaemia	3.1 (2.21, 4.34)	<0.001	2.11 (1.44, 3.09)	< 0.001
Severe anaemia	7.43 (1.44, 53.9)	0.021	2.76 (0.37, 25.04)	0.328
Ischemic heart disease	1.73 (1.31, 2.26)	<0.001	1.19 (0.86, 1.64)	0.282
Hypertension	2.02 (1.49, 2.77)	<0.001	1.39 (0.99, 1.98)	0.064
Congestive heart failure	2.84 (1.41, 5.73)	0.003	1.16 (0.49, 2.67)	0.738
Peripheral vascular disease	3.15 (1.87, 5.33)	<0.001	1.81 (0.98, 3.35)	0.056
History of stroke/transient ischemic attack	2.21 (1.5, 3.24)	<0.001	1.87 (1.2, 2.9)	0.005
Dementia	7.51 (3.45, 18.12)	<0.001	6.38 (2.75, 16.11)	< 0.001
Depression	4.77 (2.13, 11.37)	<0.001	3.82 (1.54, 9.97)	0.005
Peptic ulcer disease	2.73 (1.6, 4.64)	<0.001	1.98 (1.06, 3.67)	0.031

After adjusting for confounders, factors that were independently associated with frailty were age (adjusted OR = 1.07), female gender (adjusted OR = 1.67), DM (adjusted OR = 1.69), CKD (adjusted OR = 1.47), ESRF (adjusted OR = 3.58), history of CVA/TIA (adjusted OR = 1.87), moderate anaemia (adjusted OR = 2.11), dementia (adjusted OR = 6.38), depression (adjusted OR = 3.82), and PUD (adjusted OR = 1.98).

The mean number of comorbidities in the frail group was significantly higher at 3 (SD=1.4) compared to 2 (SD=1.3) in the non-frail group (OR 1.54 [1.41, 1.69], p<0.001) (Table 3). The frail group had almost thrice the odds of having multi-morbidity than the non-frail group (OR 2.93 [2.23, 3.87], p<0.001). The presence of multi-morbidity was significantly associated with frailty (OR 2.93, 95% CI 2.23-3.87), with overall increasing strength of association. As the number of comorbidities increased from 2 to 3, the OR significantly doubles from 2.45 (95% CI 1.43-4.47, p<0.002) to 4.85 (95% CI 2.79-8.92, p<0.001). Having more than five comorbidities increased the OR to 11.28 (95% CI 4.71-28.37, p<0.001).

**Table 3:** Correlation between multimorbidity and frailty

Number of comorbidities	Total, n (%) (N=1396)	Frail, n (%) (N=388)	Non-frail, n (%) (N=1008)	Odds ratio	95% CI	p-value
1	395 ( 28.3 )	66 ( 16.7 )	329 ( 83.3 )	1.43	(0.81, 2.65)	0.232
2	414 ( 29.7 )	106 ( 25.6 )	308 ( 74.4 )	2.45	(1.43, 4.47)	0.002
3	269 ( 19.3 )	109 ( 40.5 )	160 ( 59.5 )	4.85	(2.79, 8.92)	< 0.001
4	117 ( 8.4 )	56 ( 47.9 )	61 ( 52.1 )	6.54	(3.53, 12.69)	< 0.001
5	40 ( 2.9 )	16 ( 40 )	24 ( 60 )	4.75	(2.09, 10.91)	< 0.001
>5	31 ( 2.2 )	19 ( 61.3 )	12 ( 38.7 )	11.28	(4.71, 28.37)	< 0.001

As the study used EFS as a measure for frailty, an analysis of the domains in EFS was performed. The top five contributing domains were functional performance (93.5%), general health status (77.3%), hospital admission within the past year (70.6%), functional independence (65.2%), and medication use (62.9%). All the domains were significantly associated with frailty (p<0.001) except for social support (p=0.009).

## Discussion

In our study, we found that there is a positive correlation between multi-morbidity and frailty. As the number of comorbidities increases, the odds of frailty increase, which corroborates with the hypothesis that chronic diseases are major determinants of frailty. A recent systematic review done in community-dwelling elderly reported a pooled prevalence of about 70% for multi-morbidity and 20% for frailty [9]. In our cohort study of 1396 older surgical patients, the incidence of multi-morbidity is lower at 63.4% while the incidence of frailty is higher at 27.8%. Of note, there is also a higher proportion with an overlap of frailty and multi-morbidity - our study’s overlap was 21.9% compared to 6% in the systematic review. 

There is a lack of a gold standard measure for frailty and the definition of multi-morbidity amongst studies; hence some caution should be taken in the interpretation of this data. However, if this difference is true, it could reflect that surgical patients are frailer at baseline than community dwellers, possibly due to surgical conditions severe enough to require surgery for such as malignancies. However, it should be kept in mind that we only included patients over 70-years old, while the studies reviewed in the systemic review included patients as young as 52-years old and above.

Another observation is that only half the patients with multi-morbidity are concurrently frail. This could suggest that only a small proportion of those suffering from multi-morbidity accumulate enough biological deficits to develop frailty, putting them at higher risk than those with solely multi-morbidity or frailty. Further research into this group of patients and their surgical outcomes would be useful to tailor preventive strategies. 

Old age (adjusted OR = 1.07) and female gender (adjusted OR = 1.67) are independently associated with frailty. This is consistent with other studies that have shown frailty is more prevalent in females when age-corrected [9,11]. However, these are non-modifiable factors and can only aid in screening for the vulnerable. Of the comorbidities investigated, chronic diseases that have the strongest association are dementia, depression, ESRF, and moderate anaemia. Interestingly, comorbidities with long-term dire health consequences such as IHD, CHF, PVD, and hypertension were strongly associated with frailty on univariate analysis but after adjusting for confounding, did not contribute much to frailty, unlike mental health issues such as dementia and depression. Anaemia is one of the few potentially modifiable risk factors that can be optimised via patient blood management strategies in the relatively short lead-time up to surgery [21]. However, mitigating the risks of dementia and depression may require much more time and coordinated care from a multidisciplinary team.

Patients with dementia have complex health needs and may not keep up with other healthcare aspects such as polypharmacy and activities of daily living [22]. This group becomes more vulnerable post-surgery due to risks of postoperative cognitive dysfunction (POCD) and delirium in the recovery phase [23,24]. Dementia is associated with increased length of stay, higher complication burden, and higher six-month mortality rate [25-26]. Screening for dementia is currently not part of routine preoperative assessment at many centres. Our findings highlight the importance of incorporating dementia screening while identifying frail patients. Care can then be coordinated with specialized geriatric care teams to reduce the impact of these problems perioperatively [27].

The prevalence of depression among older adults in Singapore in the community was estimated to be 3.7%, yet the prevalence in our study was half of that at 1.8% [28]. This difference could be due to possible underdiagnosis of depression during preoperative screening or lower incidence of depression amongst health-seeking individuals who have subsequently presented for surgery. Although it has a low prevalence, depression is strongly associated with frailty (OR=3.82) and patients experience increased pain levels and poorer functional outcomes, particularly affecting long-term postoperative recovery [29,30]. Further studies are needed to investigate the association between depression, multi-morbidity, and frailty. This would provide more information to enable more targeted screening of the older patients for depression. 

Distinguishing the contributions of different domains in EFS is valuable to policymakers in deciding the resources needed to deliver tailored care. Our results show the main contributing domains were those involving a loss of function, i.e., the ability to perform activities of daily living and the number of hospital admissions in the past year. This suggests that loss of functional independence may have a disproportionate impact on the onset of frailty in older people. Deficiencies in the other domains of the EFS - availability of social support, disease burden, nutrition, continence, and mood - were less prevalent. Postoperative care and post-discharge plans into the community may benefit from focusing on returning the frail patients back to their baseline functionality. Integration of step-down medical care and rehabilitation can prevent or delay frailty by preserving functional independence.

Strengths and limitations

The strength of this study is its large sample size, collected from one of the largest tertiary hospitals in Southeast Asia, which increases its generalizability. The patients seen in the preoperative evaluation clinic represent ≥90% of the elective non-cardiac surgical load at our institution. The data is prospectively collected and has a low percentage of missing data.

Other than the intrinsic limitations of a retrospective study, another limitation of the source data is that the elective patients who are not able to attend preoperative evaluation clinic may have a different frailty and multi-morbidity profile. They may have very poor effort tolerance, or already hospitalized with repeat surgeries, or institutionalized in nursing homes and mental institutes.

## Conclusions

In conclusion, this study shows that multi-morbidity is associated with frailty with increasing strength of association as the number of comorbidities increase. Independent factors associated with frailty are age, female gender, diabetes mellitus, chronic kidney disease, end-stage renal failure, history of CVA/TIA, dementia, depression, peptic ulcer disease and moderate anaemia. Dementia and depression are comorbidities with strong associations that have yet to see coordinated interventional efforts in the preoperative clinic setting. The distribution of domains contributing to frail patients suggests that functional interventions should be targeted for prehabilitation programs.
